# Rasmussen's Aneurysm: A Rare Case

**DOI:** 10.7759/cureus.25740

**Published:** 2022-06-07

**Authors:** Swaragandha S Jadhav, Avinash Dhok, Shyam V Chaudhari, Sandeep Khandaitkar, Ashish N Ambhore

**Affiliations:** 1 Department of Radiology, NKP (Narendra Kumar Prasadrao) Salve Institute of Medical Sciences & Research Centre, Nagpur, IND; 2 Department of Physiotherapy, VSPM's College of Physiotherapy, Nagpur, IND; 3 Department of Oral and Maxillofacial Surgery, VSPM's Dental College and Research Centre, Nagpur, IND

**Keywords:** transarterial embolization, cavitary tuberculosis, tuberculosis, case report, haemoptysis, male, rasmussen’s aneurysm

## Abstract

Rasmussen’s aneurysm is a very uncommon condition occurring in post-pulmonary tuberculosis patients. We are presenting a case of a young male patient with the chief complaints of hemoptysis, breathlessness on exertion, cough with expectoration and fever, and weight loss. A thorough radiological examination revealed multiple cavitary lesions, bronchiectasis, tree-in-bud appearance and pulmonary nodules, and areas of air-spaced opacities, indicating a likely diagnosis of post-primary pulmonary tuberculosis with stages of active infection and healed infection. The post-contrast study revealed a well-defined dilated vascular channel arising from a branch of the right pulmonary artery indicating pseudo-aneurysm formation, i.e., Rasmussen’s aneurysm, within a large cavity in the right middle lobe. The patient underwent emergency trans-arterial embolization successfully and he was stable postoperatively.

## Introduction

Rasmussen’s aneurysm was first described by Fritz Valdemar Rasmussen in relation to cavitary lung lesions of tuberculosis [1]. This is a rare condition characterized by abnormal dilatation of pulmonary arteries, which occurs in about one out of 14,000 people (0.007%). Rasmussen’s aneurysm is caused due to gradual weakening of the adjacent pulmonary arterial wall. The granulation tissue replaces the adventitia and media of the pulmonary arterial wall, which in turn is replaced by fibrin, leading to thinning and pseudoaneurysm formation. The typical clinical finding in classical Rasmussen’s aneurysm is low-grade fever, night sweats, cough, and mild hemoptysis. Haemoptysis can occur in tubercular patients due to many reasons like bronchiectasis, aspergilloma, and hypervascularity from bronchial arteries but hemoptysis due to Rasmussen’s aneurysm is a rare condition that is manifested in around 8% of the population [2]. It is a life-threatening complication and needs immediate attention as it results in bleeding in the lungs. Pre and post-contrast computed tomography (CT) scans of the lung parenchyma form the basis of diagnosis [3]. This rare disorder is usually managed by local embolization technique. This case report presents a case of Rasmussen’s aneurysm in a young male.

## Case presentation

**Figure 1:**
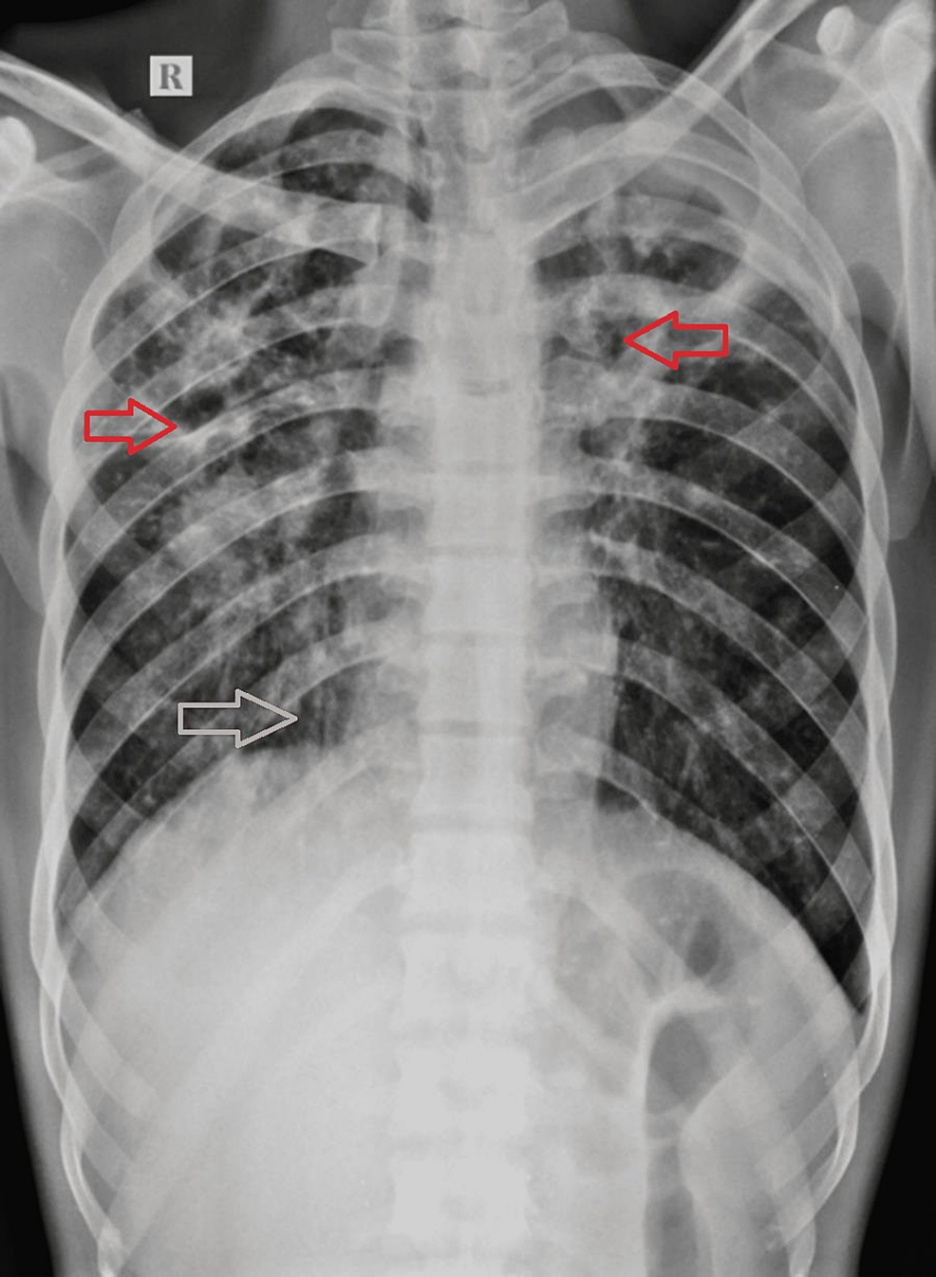
Chest x-ray posteroanterior view shows multiple cavitary lesions in bilateral upper zones and right mid-zone. Air-spaced opacities scattered throughout the bilateral (Right > Left) lung parenchyma. Tractional bronchiectatic changes in the right upper and lower lung zones. Red arrow indicates cavity at bilateral upper zone whereas white arrow indicates tractional bronchiectasis in right lower lung zone.

A young male patient was diagnosed with pulmonary tuberculosis two years ago and he was on anti-tubercular treatment category II; he had various symptoms like fever, cough with expectoration, breathlessness on exertion, and weight loss for four years, which escalated in the last seven days. The patient also complained of hemoptysis, which according to him persisted for the last four days. The patient had normal bowel and bladder habits. He was pale on general examination with multiple palpable enlarged cervical lymph nodes. No cyanosis, clubbing, icterus, or pedal edema was seen. Systemic examination was within normal limits. Emergency transarterial embolization was done. On laboratory investigations, hemoglobin was 9gm/dL, i.e. reduced, and WBC count was 14,000/mm3, i.e. raised. The rest of the laboratory investigations were within normal limits. The patient was advised for a chest x-ray, which on further evaluation revealed multiple cavitary lesions in bilateral upper lung zones and right middle lung zone with a shift of the trachea towards the right side. Multiple air-spaced radio-opacities were noted scattered throughout the bilateral (Right > Left) lung parenchyma. Tractional bronchiectatic changes were noted in the right lung parenchyma (Figure 1).

**Figure 2:**
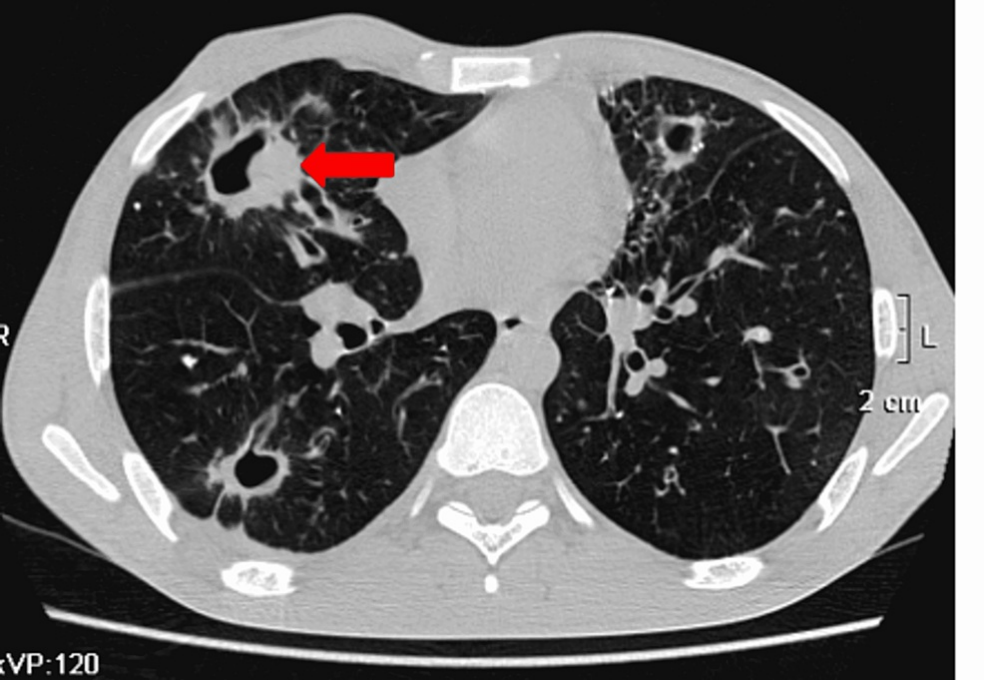
Plain CT axial section reveals multiple cavitary lesions throughout lung parenchyma.

These features were suggestive of infective etiology likely to be post-pulmonary tuberculosis. The patient was further advised cartridge-based nucleic acid amplification test (CBNAAT) and the patient came positive for tuberculosis. He was advised contrast CT chest. Plain CT axial section revealed multiple cavitary lesions throughout bilateral (Right > Left) lung parenchyma. Multiple areas of bronchiectasis, tree-in-bud appearance, and pulmonary nodules were noted in the bilateral lower lobe (Right >> Left). Areas of air-spaced opacities were noted in bilateral lower lobes (Figure 2).

**Figure 3:**
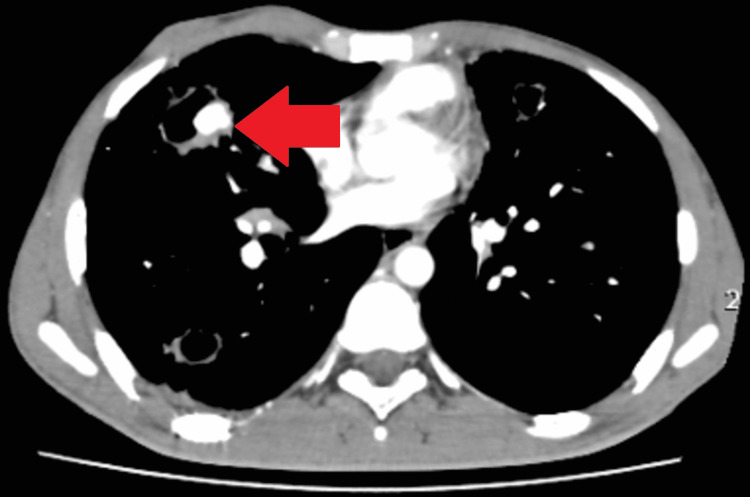
On the post-contrast study, the arterial phase axial section shows a well-defined dilated vascular channel arising from the branch of the right pulmonary artery within a large cavity in the right middle lobe with homogenous intense enhancement similar to the aorta in the arterial phase image.

In the post-contrast study, arterial phase, axial section, shows a well-defined dilated vascular channel arising from the branch of the right pulmonary artery within this soft tissue of size approximately 10.7 x 14 x 12.3 mm with homogenous intense enhancement similar to the aorta in arterial phase images. This represents pseudo-aneurysm formation, i.e. Rasmussen’s aneurysm, within a large cavity in the right middle lobe (Figure 3).

Few necrotic mediastinal paratracheal lymph nodes were noted. Pleural thickening was noted along the right lower lobe’s posterior basal segment. The above imaging features were suggestive of chronic infective etiology, likely post-pulmonary tuberculosis with stages of active infection, and healed infection with post-tubercular complication in the form of rupture of Rasmussen’s aneurysm. He was taken to the operation theatre (OT) for trans-arterial embolization. The pseudoaneurysm formed by the branch of the right pulmonary artery was embolised using polyvinyl alcohol. Embolization was successful. The procedure went uneventful. The patient showed clinical improvement with stable vital signs and thus got discharged after two days of monitoring. The patient was advised antitubercular treatment and tranexamic acid tablets that would lead to clot formation and therefore prevent bleeding on post-operative days. Post-procedure patient showed remarkable recovery. On the advised three-week follow-up, the patient had recovered well symptomatically without any complications and signs of recurrence.

## Discussion

In a developing country, low immunity is the main reason for the development of tuberculosis along with chronic immunomodulatory drug treatments, low weight, and alcohol abuse as predisposing risk factors [4].

Mild and moderate hemoptysis secondary to Rassmeusen's aneurysm is usually controlled by antitubercular therapy but massive hemoptysis usually of arterial origin needs urgent attention in the form of trans-arterial embolization [5].

Tuberculosis is a chronic inflammatory condition. Bronchial-pulmonary and arterio-venous communications are prone to rupture in the form of the formation of pseudoaneurysms. They occur secondary to the hypertrophy of bronchial arteries. Rasmussen aneurysm is a pulmonary artery pseudoaneurysm. Inflammation causes the weakening of the vessel wall. Tunica media and tunica adventitia of the pulmonary artery vessels are replaced by fibrin leading to a weakening of the wall and the formation of pseudoaneurysm [6]. Presentation of rupture of the Rasmussen’s aneurysm is in the form of hemoptysis in tubercular patients. The inflammatory degradation of the media in segmental pulmonary arteries causes hemoptysis from a Rasmussen aneurysm complicating active tuberculosis.

Bronchoscopy and CT scan are the investigations performed. CT pulmonary angiography is the imaging modality of choice. It also accurately differentiates the source of bleeding occurring from a bronchial artery or systemic artery and further guides the therapy. Transarterial embolization using gel foams, trans-arterial coiling, or use of stent-graft are the treatment modalities of choice [7]. Emergency endovascular procedures such as arterial transcatheter embolization are preferred for hemoptysis [8].

This case was considered an emergency because the patient had hemoptysis as well as a history of tuberculosis. The pseudoaneurysm formed by the branch of the right pulmonary artery was embolised using polyvinyl alcohol. This is similar to a recent study that described the case of a post-tubercular middle-aged woman who presented with a complaint of massive hemoptysis. On the contrast-enhanced CT (CECT) scan of the thorax, the lesion was diagnosed as a Rasmussen’s aneurysm within a tubercular cavity, managed by transarterial embolization successfully [9].

 A novel case of a young male was reported little while ago, who was presented with recurrent episodes of massive hemoptysis and was diagnosed with pulmonary tuberculosis. Despite being actively treated, his hemoptysis persisted. They described the role of different diagnostic modalities and the available therapeutic options to be considered in the effective management of Rasmussen’s aneurysm [10].

## Conclusions

This case report reveals an extremely rare case of Rasmussen’s aneurysm. It was diagnosed radiologically as a post-tubercular lethal complication and successfully treated by transarterial embolization of the branch of the right pulmonary artery. The present case highlights the importance of considering Rasmussen’s aneurysm in post-tubercular patients with differential diagnoses of cases reporting hemoptysis with the view to diagnose and treat the patient correctly and early to prevent fatal bleeding. This case report adds to the growing knowledge of the significance of making a diagnosis in suspected cases of hemoptysis in a post-tubercular patient, the importance of effective utilization of diagnostic aid in the form of CECT thorax, and mandatory intraoperative transarterial embolization of the feeding vessel as a management tool for Rasmussen’s aneurysm.
